# Diagnostic efficacy of large language models in the pediatric emergency department: a pilot study

**DOI:** 10.3389/fdgth.2025.1624786

**Published:** 2025-07-01

**Authors:** Francesco Del Monte, Roberta Barolo, Maria Circhetta, Angelo Giovanni Delmonaco, Emanuele Castagno, Emanuele Pivetta, Letizia Bergamasco, Matteo Franco, Gabriella Olmo, Claudia Bondone

**Affiliations:** ^1^ Department of Pediatric Emergency, Regina Margherita Children’s Hospital—A.O.U. Città Della Salute e Della Scienza di Torino, Turin, Italy; ^2^ Department of Public Health and Pediatrics, Postgraduate School of Pediatrics, University of Turin, Turin, Italy; ^3^ Department of Control and Computer Engineering, Politecnico di Torino, Turin, Italy; ^4^ Division of Emergency Medicine and High Dependency Unit, Department of Medical Sciences, Città Della Salute e Della Scienza di Torino and University of Turin, Turin, Italy; ^5^ LINKS Foundation, Turin, Italy; ^6^ Department of Clinical and Biological Sciences, University of Turin, Orbassano, Turin, Italy

**Keywords:** artificial intelligence, chatbot, diagnostic accuracy, large language model, pediatric emergency department

## Abstract

**Background:**

The Pediatric Emergency Department (PED) faces significant challenges, such as high patient volumes, time-sensitive decisions, and complex diagnoses. Large Language Models (LLMs) have the potential to enhance patient care; however, their effectiveness in supporting the diagnostic process remains uncertain, with studies showing mixed results regarding their impact on clinical reasoning. We aimed to assess LLM-based chatbots performance in realistic PED scenarios, and to explore their use as diagnosis-making assistants in pediatric emergency.

**Methods:**

We evaluated the diagnostic effectiveness of 5 LLMs (ChatGPT-4o, Gemini 1.5 Pro, Gemini 1.5 Flash, Llama-3-8B, and ChatGPT-4o mini) compared to 23 physicians (including 10 PED physicians, 6 PED residents, and 7 Emergency Medicine residents). Both LLMs and physicians had to provide one primary diagnosis and two differential diagnoses for 80 real-practice pediatric clinical cases from the PED of a tertiary care Children's Hospital, with three different levels of diagnostic complexity. The responses from both LLMs and physicians were compared to the final diagnoses assigned upon patient discharge; two independent experts evaluated the answers using a five-level accuracy scale. Each physician or LLM received a total score out of 80, based on the sum of all answer points.

**Results:**

The best performing chatbots were ChatGPT-4o (score: 72.5) and Gemini 1.5 Pro (score: 62.75), the first performing better (*p* < 0.05) than PED physicians (score: 61.88). Emergency Medicine residents performed worse (score: 43.75) than both the other physicians and chatbots (*p* < 0.01). Chatbots' performance was inversely proportional to case difficulty, but ChatGPT-4o managed to match the majority of the correct answers even for highly difficult cases.

**Discussion:**

ChatGPT-4o and Gemini 1.5 Pro could be a valid tool for ED physicians, supporting clinical decision-making without replacing the physician's judgment. Shared protocols for effective collaboration between AI chatbots and healthcare professionals are needed.

## 1 Introduction

Large Language Models (LLMs) are advanced artificial intelligence (AI) systems that understand and generate natural language (1). Among the most popular ones, OpenAI's GPT models (2) such as Chat Generative Pre-trained Transformer (ChatGPT) (3), Google's Gemini series (4), and Meta's LLaMA family (5), gained attention in the open-source community. These models are trained on vast amounts of textual data, and their performance improves as the quantity and quality of training data increase (1).

LLMs can be applied in clinical decision support, medical record analysis, patient engagement, and dissemination of health information (6). AI-based tools can support healthcare professionals by offering diagnostic assistance, thereby increasing accuracy, efficiency and enhancing clinical outcomes (7, 8). However, sometimes their responses could be inaccurate or misleading, underscoring the need for rigorous validation and oversight in clinical settings (9, 10).

In 2023, Kanjee et al. (11) examined the diagnostic accuracy of ChatGPT-4, showing that AI included the correct diagnosis in differential-diagnosis lists in 64.0% of cases, successfully identifying the main diagnosis in 39.0%. In the same year, Hirosawa et al. (12) evaluated ChatGPT-3 on common clinical scenarios, showing that it included the correct diagnosis in 93.3% of differential-diagnosis lists, though physicians outperformed the model in ranking accuracy. In a follow-up study (13), the same team showed that ChatGPT-4 performed better than ChatGPT-3.5 and comparably to physicians, although the differences were not significant. Recently Hirosawa et al. (14) tested different chatbots on adult cases: ChatGPT-4 achieved the highest accuracy, including correct diagnoses in 86.7% of lists and identifying the main diagnosis in 54.6% of cases.

To our knowledge, the role of LLMs as a diagnostic support tool in the Pediatric Emergency Department (PED) has not been explored yet. In our pilot study we tested the diagnostic efficacy of some of the most used LLMs on pediatric emergency clinical vignettes and compared their performance to a group of physicians. Our aim was to evaluate whether LLMs can serve as an effective support to ED physicians in formulating accurate diagnoses for pediatric emergency clinical cases.

## 2 Materials and methods

### 2.1 Study design

This prospective observational diagnostic study was conducted at our PED between March and October 2024. Our tertiary care teaching hospital provides care for critically ill patients younger than 18 years. The study was performed according to the international regulatory guidelines and current codes of Good Epidemiological Practice.

Two experienced pediatricians created a dataset of 80 cases with varying clinical complexity, from different pediatric subspecialties (Table 1). We extracted the cases from anonymized records of children admitted to our PED between September 2018 and May 2024. We excluded trauma and cases in which the final diagnosis was reached mainly through laboratory or instrumental tests. Patients and their parents did not provide written or oral informed consent, as all the cases were anonymized before the vignettes were generated and no sensitive data was reported. Since it was not possible to trace the identity of the patients and since this study did not retrospectively influence in any way the clinical management of the cases described, the approval of the Ethics Committee was not necessary.

**Table 1:** Clinical cases divided by pediatric subspecialties.

Pediatric subspecialty	Number of cases	List of clinical cases
Respiratory system	8 (4)	Bronchiolitis, Pneumothorax, Foreign body inhalation, Pneumonia, Wheezing, Acute laryngitis, Pneumomediastinum, Whooping cough
Infectivology	17 (13)	Bronchiolitis, Thyroglossal duct infection, Acute otitis media, Periorbital cellulitis, Pneumonia, Group A beta hemolytic Streptococcus acute pharyngotonsillitis, Otomastoiditis, Pyelonephritis, Retropharyngeal abscess, Malaria, Mononucleosis, Staphylococcal Scalded Skin Syndrome, Osteomyelitis, Meningoencephalitis, Pertussis, Staphylococcal toxic shock syndrome, Acute laryngitis
Orthopedics	7 (2)	Painful pronation of the elbow, Transient synovitis of the hip, Legg-Calvé-Perthes disease, Epiphysiolysis, Griesel's syndrome, Osteomyelitis, Osteosarcoma
Ear-Nose-Throat (ENT)	5 (4)	Thyroglossal duct infection, Acute otitis media, Laryngomalacia, Retropharyngeal abscess, Otomastoiditis
Gastroenterology	8 (1)	Appendicitis, Intestinal intussusception, Inflammatory bowel disease, Cyclic vomiting syndrome, Biliary tract atresia, Hirschsprung's disease, Functional abdominal pain, Alagille's syndrome
Oncology	4 (2)	Osteosarcoma, Leukemia, Central nervous system tumor (2 cases, different clinical presentation)
Endocrinology	3 (0)	Onset of diabetes mellitus type 1, Hypothyroidism, Addison's disease
Haematology	7 (2)	Immune thrombocytopenia, Post-infectious bone marrow aplasia in patient with spherocytosis, Haemophilia, Post-infectious acute hemolytic anaemia, Acute haemolytic crisis in favism, Retinal thrombosis in autoimmune disease, Haemolytic-uremic syndrome
Nephrology	4 (2)	Post-infectious glomerulonephritis, Pyelonephritis, Idiopathic nephrotic syndrome, Haemolytic-uremic syndrome
Immunology and rheumatology	7 (4)	Kawasaki syndrome, Sydenham's chorea, Rheumatic disease, Systemic juvenile idiopathic arthritis, Schoenlein-Henoch purpura, Ataxia telangiectasia, Retinal thrombosis in autoimmune disease
Neurology	17 (6)	Guillain-Barré syndrome, Charcot-Marie-Tooth disease, Conversion disorder, Transverse myelitis, Febrile seizures, Trigeminal neuralgia, Central nervous system demyelinating disease, Gastroenteritis-associated seizures, Migraine with aura, Iatrogenic peripheral neuropathy, Meningoencephalitis, Central nervous system tumor (2 cases, different clinical presentation), Peripheral paralysis of the VII cranial nerve, Ataxia telangiectasia, Narcolepsy, Sydenham's chorea
Allergology	3 (0)	Cow's milk protein allergy, Food Protein-Induced Enterocolitis Syndrome, Anaphylactic shock
Cardiology	5 (2)	Complete atrioventricular block in rare pathology (KSS), Myocarditis/heart failure, Vaso-vagal syncope, Rheumatic disease, Alagille's syndrome
Dermatology	4 (3)	Staphylococcal Scalded Skin Syndrome, Subgaleal hematoma, Kwashiorkor, Staphylococcal toxic shock syndrome
Dietetics and Nutrition	2 (1)	Scurvy, Kwashiorkor
Genetics	2 (2)	Alagille's syndrome, Charcot-Marie-Tooth disease
Toxicology	2 (0)	Acute accidental intoxication by cannabinoids, Methaemoglobinemia due to local anesthetic
Ophthalmology	1 (1)	Retinal thrombosis in autoimmune disease

If the case involved more than one medical subspecialty, it was included in all categories and was underlined in the table. The number of cases for each subspecialty is reported in the second column as the total number (number of cases referring to more than one subspecialty). In the right column, the underlined diagnoses are those referring to more than one subspecialty.

**Figure 1:**
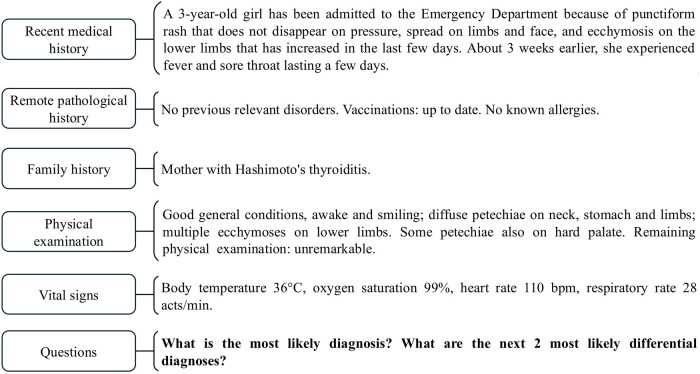
Example of a clinical vignette translated in English, subdivided into its main parts.

Each case was used to generate a clinical vignette written in Italian by the two main investigators. The clinical vignettes were prepared both to be input as a prompt to different LLM-based chatbots and to be evaluated by a group of physicians. In each vignette (Figure 1), we presented all the main details as follows: recent and past medical history, relevant family medical history, physical examination and vital signs. Laboratory tests were not reported.

The vignettes were submitted to a panel of three independent expert pediatricians who validated the cases or recommended a revision. They also independently ranked them according to three levels (lowly difficult, difficult, and highly difficult), based on solving complexity according only to available clinical data. The final level for each case was determined based on the majority agreement among the experts: 20 (25.00%) highly difficult, 31 (38.75%) difficult, and 29 (36.25%) lowly difficult.

The two main investigators evaluated all the answers generated by LLMs and physicians, and statistical analysis was performed.

### 2.2 LLM-based chatbots answers

We selected four of the highest rated (15, 16) LLMs publicly available during the period in which this study was conducted: ChatGPT-4o (17) and ChatGPT-4o mini (18) (OpenAI); Gemini 1.5 Flash (19) and Gemini 1.5 Pro (19) (Google); and Llama-3-8B (20) Instruct version (Meta), an open-source model satisfying our computational resources constraints. Unlike the other LLMs, which were used through the web interface, Llama-3-8B was deployed in our computing infrastructure and could be used without requiring internet access. The characteristics and access details of the selected LLMs are summarized in Table 2 (3, 25–27).

**Table 2:** LLM-based chatbots selected for the study and their access details.

Chatbot access details	ChatGPT-4o (3)	ChatGPT-4o mini (3)	Gemini 1.5 Flash (25)	Gemini 1.5 Pro (26)	Llama-3-8B (27)
Provider	OpenAI	OpenAI	Google	Google	Meta
Access date	August 15, 2024	August 26, 2024	August 21, 2024	August 19–20, 2024	August 27, 2024
Open-source	No	No	No	No	Yes
Free (at the time of this study)	No (free questions available up to a daily limit)	Yes, after login	Yes, after login	No (Free questions available up to a daily limit)	Yes

The chatbots were not provided with any example of the task at hand. Moreover, each vignette was given as a prompt to each chatbot only once in independent chats, to prevent LLMs from applying any learning and inference to subsequent cases. At the end of each vignette, we asked two open-ended questions: “What is the most likely diagnosis? Which are the next two more likely differential diagnoses?”.

### 2.3 Physicians' answers

Twenty-three physicians were selected to evaluate the clinical cases, including 10 PED physicians with at least 5 years of experience, 6 residents attending their last year of residency in Pediatrics at our PED, and 7 residents attending their last year of residency in Emergency Medicine (EM) at the University of Turin, Italy. These three subgroups were selected to ensure a diverse range of clinical experiences and perspectives. The main investigators were excluded.

Between July and August 2024, the participants were asked to resolve the 80 vignettes through Google Forms. The use of digital resources, textual assistance or consulting colleagues were forbidden, to ensure that the responses were purely the result of the physician's independent clinical reasoning and experience.

The vignettes were presented in random difficulty order and divided in 4 standardized forms with 20 cases each, in order to minimize the risk of fatigue for participants, thus influencing the quality of responses. As for chatbots, we asked the same questions to physicians for each vignette.

### 2.4 Evaluation method

The answers obtained from LLMs and physicians were independently evaluated by the two main investigators and compared to the final diagnoses established at the time of patients' discharge from the PED or following hospitalization. Each answer was evaluated through a 5-point accuracy scale, in order to avoid penalizing incomplete or imprecise diagnoses that still demonstrated adequate clinical reasoning: 1 (correct main diagnosis); 0.75 (if the correct diagnosis was identified within differential diagnoses); 0.5 (if the main diagnosis was correct, but not precise); 0.25 (if the correct diagnosis was identified within differential diagnoses, but not precise); and 0 (both main and differential diagnoses were incorrect).

In case of disagreements between the two main investigators, they reached consensus facing each other. Each physician or LLM received a total score by summing the points obtained from all answers, thus obtaining 80 as the maximum possible score.

### 2.5 Statistical analysis

Descriptive statistics were presented using mean ± standard deviation (SD), and median and Interquartile Range (IQR) to report the performance of LLMs and physicians, as appropriate. Bar charts, stacked charts, and dot plots were used to visualize the total scores obtained by the different groups and comparison. For the statistical analysis, a long-format dataset was created. The distribution of accuracy count was checked using histograms and Q-Q plots. Comparisons between physicians and LLMs were made using the Kruskal–Wallis H test. Pairwise comparisons were made using Dunn's procedure with Bonferroni correction for multiple comparisons. Multinomial logistic regression models were used to assess the probability of correct response (accuracy = 1) of physician groups and chatbots by difficulty of the cases. Adjusted predicted probabilities of scoring one in accuracy and their 95% confidence intervals were estimated for each difficulty level and group. The results were reported using a line plot with error bars. Statistical significance was set at *p* < 0.05. Analyses were conducted using STATA 18.5.

## 3 Results

Overall, we obtained a total of 1,840 responses from the 23 physicians (800 from PED physicians, 480 from PED residents, 560 from EM residents) and 400 responses from the 5 selected chatbots.

**Figure 2:**
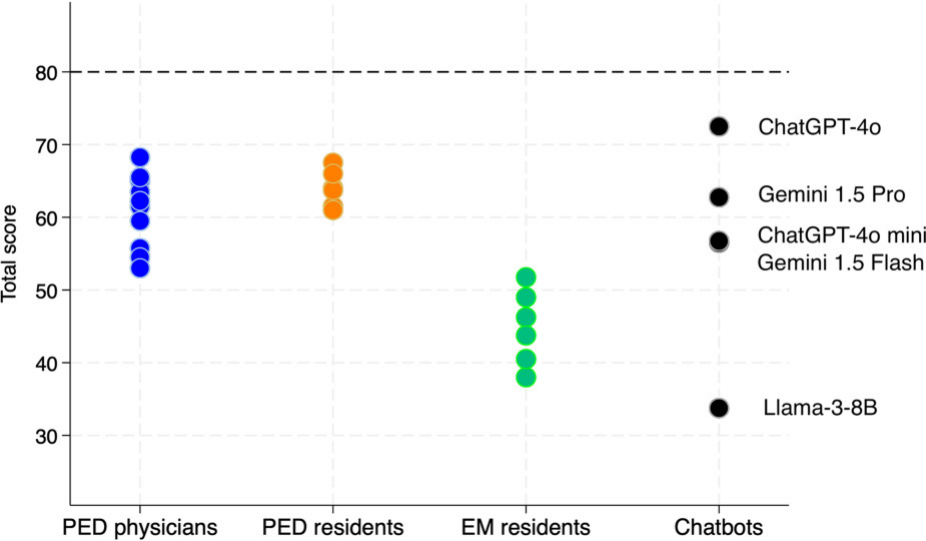
Total scores for each evaluator, grouped by category. PED, pediatric emergency department; EM, emergency medicine.

The highest and lowest total accuracy scores were obtained respectively by ChatGPT-4o (72.5) and Llama-3-8B (33.75). Gemini 1.5 Flash, ChatGPT-4o mini and Gemini 1.5 Pro scored 56.5, 56.75, and 62.75, respectively. PED physicians (60.88 ± 4.83) and PED residents (63.96 ± 2.3) achieved the highest scores, followed by EM residents (44.25 ± 4.64) (Figure 2; Supplementary Material Table 1).

**Figure 3:**
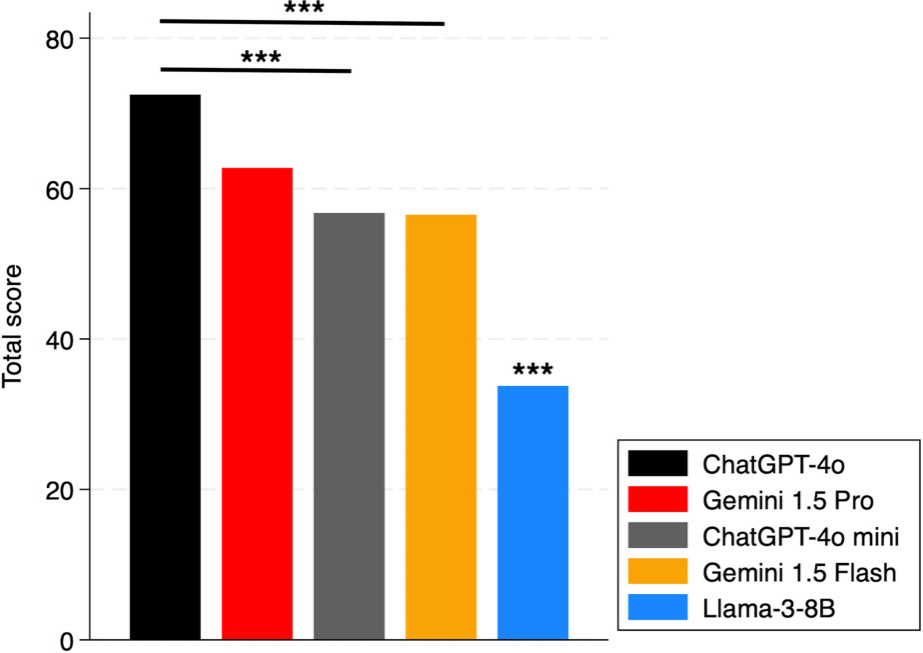
Total scores of chatbots. The *** above the bar shows the *p*-values of the comparisons of that subject vs. all others. ***: *p* < 0.01.

As regards chatbots, significant difference was found between the total accuracy performance of ChatGPT-4o and ChatGPT-4o mini (*p* < 0.01), and between ChatGPT-4o and Gemini 1.5 Flash (*p* < 0.01). Llama-3-8B performed worse than all the other chatbots (*p* < 0.01). No difference was observed between ChatGPT-4o and Gemini 1.5 Pro (*p* = 0.26) (Figure 3).

**Figure 4:**
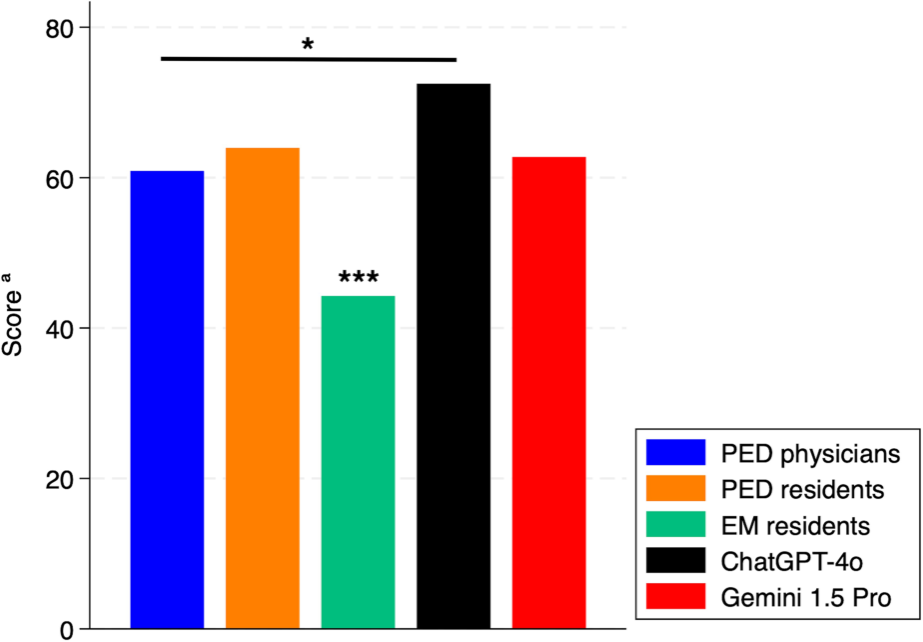
Total scores of chatbots and physician subgroups. PED, pediatric emergency department; EM, emergency medicine. ^a^Median of total score for physicians. *: *p* < 0.05. ***: *p* < 0.01.

Comparing the median total scores of physicians to the single performance of the best performing chatbots (ChatGPT-4o and Gemini 1.5 Pro) (Figure 4), we observed no significant difference between PED residents and chatbots. However, ChatGPT-4o performed better than PED physicians (*p* < 0.05), while EM residents performed worse than both the other physicians and chatbots (*p* < 0.01).

**Figure 5:**
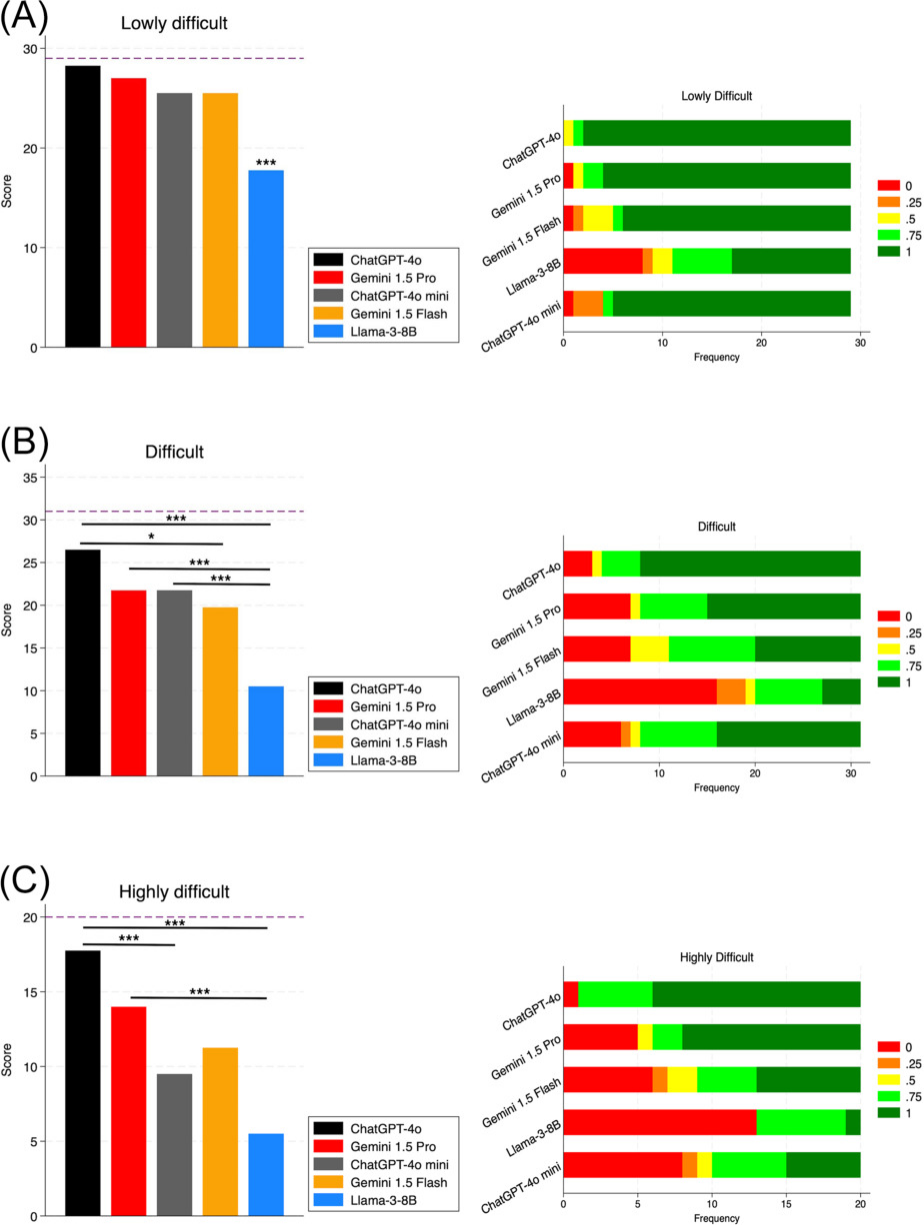
Chatbots’ diagnostic performance by case difficulty. Panels on the left show the total scores for each chatbot; panels on the right show the frequency of accuracy levels achieved. **(A)** Lowly difficult cases; **(B)** difficult cases; **(C)** highly difficult cases. The dashed line shows the maximum obtainable total score for the specific difficulty level. *: *p*-value < 0.05. ***: *p*-value < 0.01.

In lowly difficult cases, all chatbots but Llama-3-8B performed well; Llama-3-8B showed a significant difference compared to other chatbots (*p* < 0.01). In difficult cases, ChatGPT-4o performed better than Gemini 1.5 Flash (*p* < 0.05) and Llama-3-8B (*p* < 0.01). Gemini 1.5 Pro and ChatGPT-4o mini performed better than Llama-3-8B (*p* < 0.01). We did not find any significant between ChatGPT-4o and Gemini 1.5 Pro and between Gemini 1.5 Flash and Llama-3-8B. As regards highly difficult cases, ChatGPT-4o performed significantly better than ChatGPT-4o mini (*p* < 0.01) and Llama-3-8B (*p* < 0.01); also Gemini 1.5 Pro performed significantly better than Llama-3-8B (*p* < 0.01) (Figure 5). ChatGPT-4o showed not only higher performance, but also better accuracy (Figure 5C), providing completely incorrect answers only in 4/80 cases (3 difficult, 1 highly difficult).

**Figure 6:**
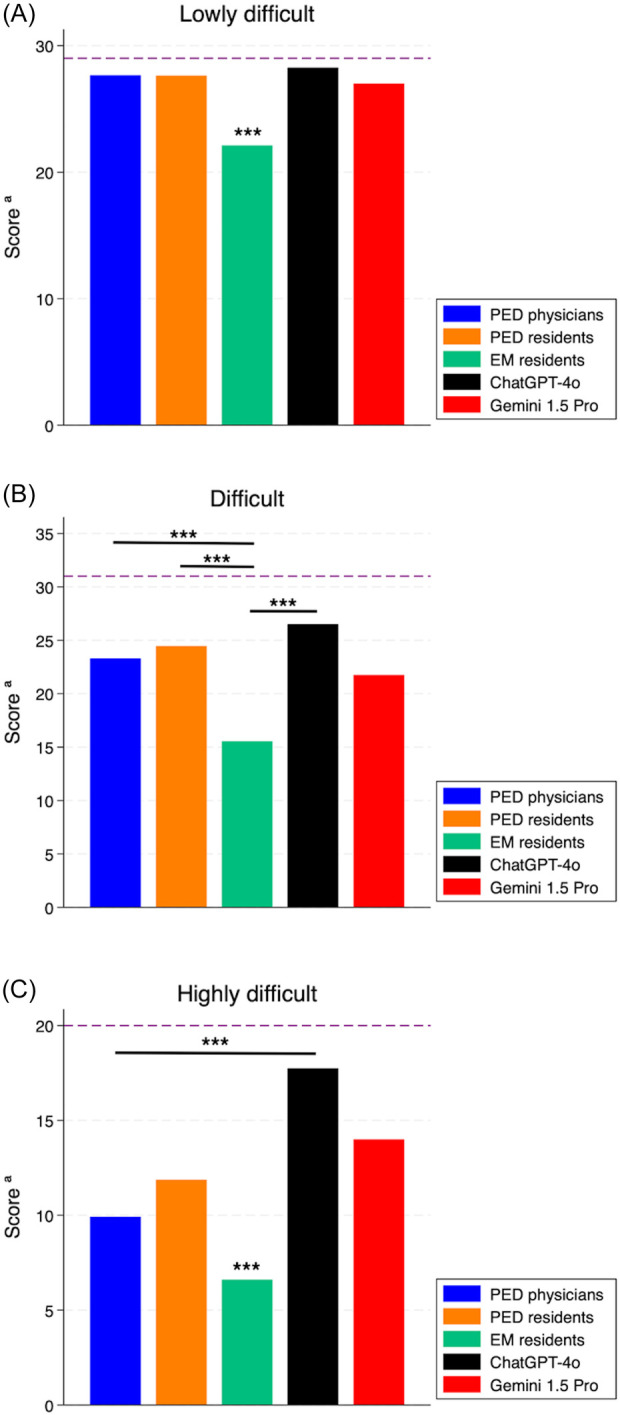
Score of the best performing chatbots (ChatGPT-4o and Gemini 1.5 Pro) compared to the median score obtained from physician subgroups, stratified by case difficulty. **(A)** Lowly difficult cases; **(B)** difficult cases; **(C)** highly difficult cases. PED, pediatric emergency department; EM, emergency medicine. The dashed line shows the maximum obtainable score for the specific difficulty level. *: *p*-value < 0.05. ***: *p*-value < 0.01.

Last, we compared the two best performing chatbots (ChatGPT-4o and Gemini 1.5 Pro) to the median score obtained from the subgroups of physicians, stratified by difficulty (Figure 6). As regards the lowly and highly difficult cases, PED physicians, PED residents and both chatbots performed significantly better than EM residents (*p* < 0.01). In difficult cases, PED physicians, PED residents and ChatGPT-4o performed significantly better than EM residents (*p* < 0.01), but not Gemini 1.5 Pro (*p* > 0.05). In highly difficult cases, both ChatGPT-4o and Gemini 1.5 Pro performed better than PED physicians and PED residents; however, statistical significance was reached only in the comparison between ChatGPT-4o and PED physicians (*p* < 0.01).

**Figure 7:**
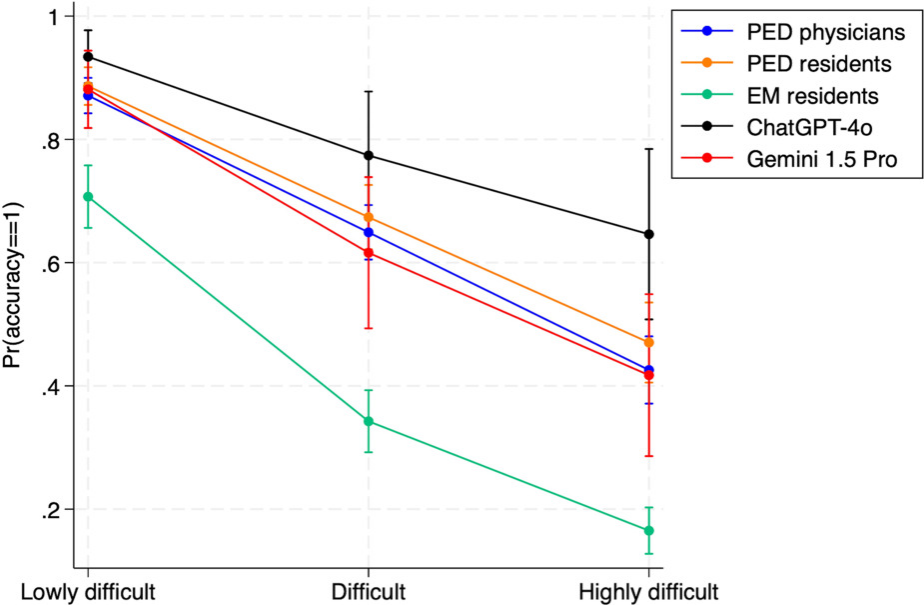
Adjusted predictions and their 95% confidence intervals of the probability of identifying the correct answer of the vignettes in the main diagnosis (accuracy = 1) for subgroups of physicians, ChatGPT-4o and Gemini 1.5 Pro, stratified by difficulty. PED, pediatric emergency department; EM, emergency medicine.

Figure 7 illustrates the adjusted predictions and their 95% confidence interval for the probability of giving the right answer in the “main diagnosis”, stratified by vignette difficulty. The adjusted prediction of the probability of obtaining the highest accuracy score (score = 1) was very close across all levels of difficulty for PED physicians and PED residents, with their respective confidence intervals overlapping. EM residents showed lower probability of obtaining the maximum level of accuracy than both PED physicians and residents groups, and chatbots in all levels of difficulty. ChatGPT-4o showed marginally better probability prediction than Gemini 1.5 Pro and the other groups, particularly for highly difficult cases. However, due to the single imputation, it retained broad confidence intervals.

Moreover, Table 3 shows the obtained estimates of the multinomial logistic regression, i.e., the Relative Risk Ratio (RRR) for the probability of scoring 1 (vs. 0) by evaluators. Given the same difficulty, EM residents showed a 76% lower probability of scoring 1 (vs. 0) than PED physicians. Furthermore, ChatGPT-4o had a 376% higher probability of scoring 1 (vs. 0) than PED physicians. However, the estimates show a very wide 95% confidence interval, due to the comparison between one measurement (ChatGPT-4o) vs. multiple measurements (group of PED physicians).

**Table 3:** Multinomial regression: relative risk ratio (RRR) for the probability of scoring 1 (vs. 0) by evaluators (physician groups, ChatGPT-4o, and Gemini 1.5 Pro).

Accuracy	Groups	RRR	SD	*p*-value	(95% CI)
1 vs. 0	PED physicians	1			
	PED residents	1.39	0.25	0.07	(0.98–1.97)
	EM residents	0.24	0.04	<0.01	(0.17–0.32)***
	ChatGPT-4o	4.76	2.56	<0.01	(1.66–13.67)***
	Gemini 1.5 Pro	1.04	0.37	0.90	(0.53–2.07)

Adjustment: difficulty.

*** *p* < 0.01.

## 4 Discussion

To our knowledge, this is the first study exploring the role of LLMs as diagnostic support tools in pediatric emergency cases. Among the tested chatbots, ChatGPT-4o achieved the highest accuracy, with most diagnoses aligning with correct answers for any level of complexity. In fact, ChatGPT provided a completely incorrect answer, scoring 0, in only 4 cases out of 80 (3 classified as difficult, and 1 as highly difficult). Gemini 1.5 Pro performed slightly below ChatGPT-4o, being more affected by case difficulty. Gemini 1.5 Flash and ChatGPT-4o mini achieved similar performance, but were inferior to ChatGPT-4o and Gemini 1.5 Pro: their performance was notably better in simpler cases, while it dropped in difficult and highly difficult cases. In contrast, Llama-3-8B showed significantly lower performance than all the other LLMs considered in this research. This was aligned with expectations, as it had only 8 billion parameters and the lowest scores on benchmarks (1, 15) and leaderboards (16). However, during the study period, models like Gemini 1.5 Pro and ChatGPT-4o were paid services, with free questions available up to a daily limit; this may represent a limitation for some users. On the other hand, Llama-3-8B is open-source, free, and offers greater data privacy when used on-premises, though it requires a more complex setup and adequate computational resources compared to web-based chatbots. With more computational available resources, larger models such as Llama-3-70B (20) could be tested, offering significantly more parameters and potentially better performance.

Ultimately, this study underscores the importance of human oversight in the use of LLMs, as their success in healthcare stands on accurate data collection (e.g., medical history, physical examinations and vital signs) and interpretation, which only qualified practitioners can provide. LLMs are designed to complement physicians (21), whose role is not replaceable by AI since clinical data must be evaluated by a human and then be presented to AI in the correct way, such as in terms of language, in order to be analyzed effectively and usefully. Establishing specific clinical guidelines and protocols for the use of AI in healthcare is crucial to ensure in the future the safe integration of these tools into clinical practice. Looking ahead, the integration of LLMs into PED workflows such as electronic health records or diagnostic decision support systems is a desirable goal, but remains premature at this stage. Further research is needed to assess their reliability, clinical utility, and safe implementation in real-time diagnostic settings.

Regarding the physician groups, there was no significant difference in diagnostic accuracy between PED physicians and PED residents, while a clear difference emerged between EM residents and the two pediatric physician groups. As expected, all the human subgroups showed a decline in diagnostic accuracy as case complexity increased. ChatGPT-4o and Gemini 1.5 Pro performed like PED physicians and PED residents in lowly difficult and difficult cases, and proved to be effective aids in solving highly difficult cases (e.g., rare, complex diseases).

Interestingly, ChatGPT-4o performed better than both PED residents and PED physicians, but significance was reached only vs. the latter, particularly in highly difficult cases. This observation is difficult to interpret and could be due to different physician's subgroups sample size. We can argue that PED residents performed better than PED physicians in those cases requiring knowledge of rare internal conditions, due to their more recent training. Anyway, our results cannot support this hypothesis and further investigation on a larger sample should be carried out.

All LLMs outperformed EM residents, likely due to their limited experience with pediatrics cases. In situations where a pediatrician is not immediately available, EM physicians could leverage the insights provided by LLMs alongside their own knowledge, allowing for initial diagnostic hypotheses. In the fast-paced ED environment, this could be a valuable advantage, speeding up the diagnostic process. On the other hand, our observation highlights the importance of implementing pediatric skills for EM residents, as in many cases children accessing the EDs are first evaluated by adult EM specialists, and not by specifically trained pediatricians. Pediatric skills should be not only acquired, but also maintained through longitudinal training programs during residency, as recently proposed (22).

While our study demonstrates the effectiveness of advanced LLMs in pediatric cases, a similar study by Barile et al. (23) showed significantly poorer outcomes using ChatGPT-3.5. Their investigation on 100 pediatric case challenges found a diagnostic error rate of 83%, highlighting limitations of older LLM versions. In contrast, our results indicate that state-of-art models (i.e., ChatGPT-4o and Gemini 1.5 Pro) achieved diagnostic accuracy comparable or even better than emergency pediatricians. This observation underscores the rapid advances in LLM technology and the importance of leveraging the most up-to-date tools to maximize clinical usefulness.

In fact, a general limitation when trying to evaluate LLMs performance in each context is the rapid advancement of these technologies, which can quickly make the results outdated. Moreover, LLMs are limited by the point in time when their training data are updated. If they are not fine-tuned or updated periodically, they may lack awareness of more recent data and information.

Our study has some strengths. First, we evaluated the effectiveness of the latest available versions of LLMs, ranked among the top models on the Chatbot Arena leaderboard (16) and across various benchmarks (1, 15). Such chatbots differ in model size, provider, user-interface, and availability. In contrast, many previous studies have focused on a single model, often an earlier version of ChatGPT (11–13, 23). Second, we considered three distinct groups of physicians, allowing for diverse perspectives and detailed insights in addressing the assigned tasks. Last, we introduced a non-binary evaluation approach, using multiple accuracy categories to allow for more nuanced assessments.

Our study also has some limits. First, as LLMs may show a lack of reproducibility, they could produce different responses when presented with the same case multiple times, sometimes reversing the order of diagnoses. This issue was not explored in our research.

Second, to avoid potential learning or contamination effects across prompt repetitions, each vignette was submitted only once per LLM. However, this approach prevents the assessment of intra-model variability. Future work should include repeated sampling to better quantify the consistency and stability of LLM-generated outputs. Sequential inputs or follow-up questions could also be explored, to simulate more closely real clinical conversations and evaluate their impact on diagnostic reasoning performance.

Moreover, when analyzing the physicians’ responses, we did not consider factors like a distracting environment, focus level, and stress or fatigue, which may increase inaccuracies, especially at the end of the forms. On the other hand, the process of reasoning on a clinical vignette is different from reasoning in front of a real patient: the clinical impression “at first sight” is crucial to reach the correct diagnosis and could be difficult to reproduce by written description (24). Such limitations do not affect the responses provided by chatbots.

Furthermore, the varying number of cases across difficulty levels, with only 20 cases for the hardest ones, represents a limitation. Another limit is the non-homogeneity of the number of physicians per group. This may have affected the reliability of estimates for smaller groups, as they are more sensitive to outliers. In statistical analysis, physicians' performances were summarized using the median score and compared with the absolute score of each chatbot. This difference in measurement may limit the accuracy of direct comparisons, affecting the generalizability of the results.

In conclusion, the results of our pilot study highlight the importance of understanding the diagnostic performance among different LLMs, especially in more complex PED clinical cases. Our observations suggest that certain LLMs, especially ChatGPT-4o and Gemini 1.5 Pro, have diagnostic efficacy similar to or even better than those of pediatricians. Due to their high level of accuracy, LLMs could serve as a valuable tool to support PED physicians in solving the most difficult pediatric emergency cases, and they can be a very useful tool for EM physicians for all degrees of difficulty of pediatric cases. However, LLMs should never substitute human clinical judgement.

## Data Availability

The raw data supporting the conclusions of this article will be made available by the authors, without undue reservation.
