# Correction: Diagnostic efficacy of large language models in the pediatric emergency department: a pilot study

**DOI:** 10.3389/fdgth.2025.1658635

**Published:** 2025-07-16

**Authors:** Francesco Del Monte, Roberta Barolo, Maria Circhetta, Angelo Giovanni Delmonaco, Emanuele Castagno, Emanuele Pivetta, Letizia Bergamasco, Matteo Franco, Gabriella Olmo, Claudia Bondone

**Affiliations:** ^1^ Department of Pediatric Emergency, Regina Margherita Children’s Hospital—A.O.U. Città Della Salute e Della Scienza di Torino, Turin, Italy; ^2^ Department of Public Health and Pediatrics, Postgraduate School of Pediatrics, University of Turin, Turin, Italy; ^3^ Department of Control and Computer Engineering, Politecnico di Torino, Turin, Italy; ^4^ Division of Emergency Medicine and High Dependency Unit, Department of Medical Sciences, Città Della Salute e Della Scienza di Torino and University of Turin, Turin, Italy; ^5^ LINKS Foundation, Turin, Italy; ^6^ Department of Clinical and Biological Sciences, University of Turin, Turin, Italy

**Keywords:** artificial intelligence, chatbot, diagnostic accuracy, large language model, pediatric emergency department

Affiliation “Department of Public Health and Pediatrics, Postgraduate School of Pediatrics, University of Turin, Turin, Italy” was erroneously given as “Department of Public Health and Pediatrics, University of Turin, Turin, Italy”.

The original version of this article has been updated.

