# Supplementary material for: Diagnostic efficacy of large language models in the pediatric emergency department: a pilot study
Source: Front Digit Health. 2025 Jul 1;7:1624786. doi: 10.3389/fdgth.2025.1624786 (PMC12259579; doi:10.3389/fdgth.2025.1624786)
Supplement: Supplementary file 1 [file Table1.docx]

Supplementary Material

# **Supplementary Table 1.** Accuracy scores of physicians and chatbots (individuals and groups). The maximum achievable score was 80. PED: Pediatric Emergency Department; EM: Emergency Medicine; SD: Standard Deviation; IQR: Inter-Quartile Range.

| ·· | **Absolute score** | **Mean score** | **SD** | **Median score** | **IQR** |
| --- | --- | --- | --- | --- | --- |
| **PED physician (1)** | 53 | ·· | ·· | ·· | ·· |
| **PED physician (2)** | 54.5 | ·· | ·· | ·· | ·· |
| **PED physician (3)** | 55.75 | ·· | ·· | ·· | ·· |
| **PED physician (4)** | 59.5 | ·· | ·· | ·· | ·· |
| **PED physician (5)** | 61.5 | ·· | ·· | ·· | ·· |
| **PED physician (6)** | 62.25 | ·· | ·· | ·· | ·· |
| **PED physician (7)** | 63.5 | ·· | ·· | ·· | ·· |
| **PED physician (8)** | 65 | ·· | ·· | ·· | ·· |
| **PED physician (9)** | 65.5 | ·· | ·· | ·· | ·· |
| **PED physician (10)** | 68.25 | ·· | ·· | ·· | ·· |
| **PED physicians (total)** | ·· | 60.88 | 4.83 | 61.88 | 56-65 |
| **PED resident (1)** | 61 | ·· | ·· | ·· | ·· |
| **PED resident (2)** | 61.5 | ·· | ·· | ·· | ·· |
| **PED resident (3)** | 63.75 | ·· | ·· | ·· | ·· |
| **PED resident (4)** | 64 | ·· | ·· | ·· | ·· |
| **PED resident (5)** | 66 | ·· | ·· | ·· | ·· |
| **PED resident (6)** | 67.5 | ·· | ·· | ·· | ·· |
| **PED residents (total)** | ·· | 63.96 | 2.3 | 63.88 | 62-66 |
| **EM resident (1)** | 38 | ·· | ·· | ·· | ·· |
| **EM resident (2)** | 40.5 | ·· | ·· | ·· | ·· |
| **EM resident (3)** | 40.5 | ·· | ·· | ·· | ·· |
| **EM resident (4)** | 43.75 | ·· | ·· | ·· | ·· |
| **EM resident (5)** | 46.25 | ·· | ·· | ·· | ·· |
| **EM resident (6)** | 49 | ·· | ·· | ·· | ·· |
| **EM resident (7)** | 51.75 | ·· | ·· | ·· | ·· |
| **EM residents (total)** | ·· | 44.25 | 4.64 | 43.75 | 41-49 |
| **ChatGPT-4o** | 72.5 | ·· | ·· | ·· | ·· |
| **Gemini 1.5 Pro** | 62.75 | ·· | ·· | ·· | ·· |
| **ChatGPT-4o mini** | 56.75 | ·· | ·· | ·· | ·· |
| **Gemini 1.5 Flash** | 56.5 | ·· | ·· | ·· | ·· |
| **Llama-3-8B** | 33.75 | ·· | ·· | ·· | ·· |
| **Chatbots (total)** | ·· | 56.45 | 12.76 | 56.75 | 57-63 |
